# MicroRNA-140-3p represses the proliferation, migration, invasion and angiogenesis of lung adenocarcinoma cells via targeting TYMS (thymidylate synthetase)

**DOI:** 10.1080/21655979.2021.2009422

**Published:** 2021-12-16

**Authors:** Shanzhi Wan, Zhimin Liu, Yang Chen, Zhitao Mai, Mingming Jiang, Qingguo Di, Baohua Sun

**Affiliations:** a No.1 Department of Respiratory and Critical Care Medicine, Cangzhou Central Hospital, No. 16, Xinhua West Road, Yunhe District, Cangzhou City, Hebei Province, China; b Department of No. 1 Pediatrics, Cangzhou Hospital of Integrated TCM-WM, Cangzhou City, Hebei Province, China

**Keywords:** Lung adenocarcinoma, microRNA-140-3p, proliferation, metastasis, angiogenesis, thymidylate synthetase

## Abstract

MicroRNA (miR)-140-3p has been proved to repress lung adenocarcinoma (LUAD), and our study aims to further evaluate the mechanism. Bioinformatic analyses were performed. The viability, proliferation, migration, invasion and angiogenesis of transfected LUAD cells were all determined via Cell Counting Kit-8, colony formation, Scratch, Transwell, and tube formation assays. The targeting relationship between miR-140-3p and thymidylate synthetase (TYMS) was confirmed by dual-luciferase reporter assay. Relative expressions of miR-140-3p, TYMS, epithelial-to-mesenchymal transition- (E-cadherin, N-cadherin, vimentin), angiogenesis- (vascular endothelial growth factor (VEGF)), and apoptosis-related factors (cleaved caspase-3, B-cell lymphoma-2 (Bcl-2), Bcl-2-associated X protein (Bax)) were quantified by quantitative real-time polymerase chain reaction or Western blot. TYMS was high-expressed yet miR-140-3p was low-expressed in LUAD cells. Upregulation of miR-140-3p inhibited TYMS expression, viability, colony formation, migration, invasion, and tube length within LUAD cells, while downregulation of miR-140-3p did oppositely. Silenced TYMS, the downstream target gene of miR-140-3p, reversed the effects of miR-140-3p downregulation on TYMS expression, cell viability, colony formation, migration, invasion, and tube length as well as the metastasis-, apoptosis- and angiogenesis-related proteins in LUAD cells. Upregulation of miR-140-3p inhibited the proliferation, migration, invasion and angiogenesis of LUAD cells via targeting TYMS.

## Introduction

Currently, lung cancer is identified as the leading cause of morbidity and mortality worldwide, and approximately 80–85% of lung cancers have been discovered and defined as non-small cell lung cancers (NSCLCs), of which lung adenocarcinoma (LUAD), together with squamous cell carcinoma (SqCC), has emerged as the most common subtypes [1,2]. Despite efforts and achievements in the understanding of lung cancer pathogenesis, LUAD is still one of the most aggressive and rapidly fatal tumor types, posing a huge economic and social burden [3]. Therefore, it is of significance to further delve into the mechanisms of its carcinogenesis and to provide a potential therapeutic target, hoping to work out a novel therapeutic method for patients diagnosed with LUAD.

Over 90% of human transcripts have limited protein coding capacity, while they are capable of encoding non-coding RNAs, including microRNAs (miRNAs), long non-coding RNAs (lncRNAs) and circRNAs. It has been reported that circRNA-002178 could be used as a potential noninvasive biomarker for the LUAD detection and act as a target of immune therapy [4]. miRNAs belong to the family of small non-coding RNAs, which have a negatively regulatory effect on gene expressions and participate in several cellular processes, like cell cycle, cell differentiation, and cell death, as well as tumor resistance to therapeutic agents [5–7]. Recent discoveries have also unveiled the expression profiles of miRNAs in the discovery of possible theragnostic biomarkers for LUAD [8]. miR-21 expression in lung fibroblasts can trigger the trans-differentiation of fibroblast into the cancer-associated fibroblasts (CAFs), thus supporting the progression of LUAD [9]. Also, miR-182-5p possesses a regulatory effect on both chemosensitivity and the hedgehog signaling pathway of cisplatin-resistant LUAD cells via targeting GLI family zinc finger 2 (GLI2) [10]. Furthermore, significantly downregulated miR-576-3p that is evidenced in LUAD was revealed to be capable of repressing the migration and invasion of LUAD cells via targeting Serum/glucocorticoid regulated kinase 1 (SGK1) [11]. circ_0008274 upregulates granulin to promote the progression of hepatocellular carcinoma via sponging miR-140-3p [12]. miR-140-3p enhances cisplatin sensitivity and attenuates stem cell-like properties through repressing Wnt/β-catenin signaling in LUAD cells [13]. It is additionally proved that circ-AASDH functions as the progression of early-stage LUAD by targeting miR-140-3p [14]. However, its specific functions of miR-140-3p in LUAD remain to be further elaborated in detail.

Located on the short arm of chromosome 18 (18p11.32), thymidylate synthetase (TYMS) has been documented to be pivotal in the DNA biosynthesis, making it essential for the survival of cells [15,16]. Increasing evidence has suggested that TYMS continues as a critical therapeutic target for several cancer drugs, in addition to the discoveries that prove the participation of TYMS in different cancers, including LUAD [17–19]. According to starBase prediction, miR-140-3p has a targeting relationship with TYMS. However, its interaction with microRNAs, miR-140-3p to be exact, in LUAD remains poorly understood. Hence, we mainly concentrate on discovering and discussing the roles and functions of miR-140-3p and TYMS in LUAD, with the hope to work out a possible treatment strategy for LUAD in future clinical practice.

## Materials and methods

### Bioinformatic analyses

To sort the candidate gene for our study, LUAD-associated mRNA expression profile was available and downloadable from The Cancer Genome Atlas (https://www.genome.gov/Funded-Programs-Projects/Cancer-Genome-Atlas), and differential expression analysis on the retrieved data was performed in limma package by R software (v. 3.5.1, Boston, MA, USA). Meanwhile, the expression profile of miR-140-3p in LUAD was confirmed using starBase (http://starbase.sysu.edu.cn/), and possible miRNA which could target was predicted with the help of starBase (http://starbase.sysu.edu.cn/).

### Cell culture and transfection

Human bronchial epithelial cells BEAS-2B (iCell-h023), and LUAD cells H23 (iCell-h150), H358 (iCell-h159), H1299 (iCell-h153), H1395 (iCell-h154), A549 (iCell-z069), and PC-9 (iCell-h263) were ordered from iCell (Shanghai, China). Another LUAD cell line H1435 (BNCC283007) was purchased from BeiNa Bio (Beijing, China). Then, all cells were maintained in RPMI-1640 medium (90022, Solarbio, Beijing, China) blended with 10% fetal bovine serum (FBS, S9020, Solarbio, China) and 1% penicillin–streptomycin (P1400, Solarbio, China). As the expression of miR-140-3p was the highest in H23 cell and the lowest in A549 cells, these two cells were utilized for subsequent studies. Human umbilical vein endothelial cells (HUVECs, catalog no. CP-H082) used for tube formation assay were obtained from Procell (Wuhan, China) and cultured in endothelial cell growth medium-2 (EGM-2, C-22011, PromoCell, Heidelberg, Germany) containing 5% FBS and 100 U/mL-100 mg/mL penicillin-streptomycin. All cells were finally incubated in CellXpert C170 incubator (2231000759, Eppendorf, Hamburg, Germany) at 37°C with 5% CO_2_.

Small-interfering RNA against TYMS (siTYMS, SR309484) and the negative control (siNC, SR30002) were available from Origene (Rockville, MD, USA). In addition, miR-140-3p mimic (miR10004597-1-5) and inhibitor (miR20004597-1-5) were synthesized and bought from RiboBio (Guangzhou, China), and their NC (HMC0002) was obtained from Sigma-Aldrich (St Louis, MO, USA). Sequences for transfection are listed in Table 1.

**Table 1.** Sequences for transfection

Gene	Sequence (5ʹ–3ʹ)
miR-140-3p mimic	UACCACAGGGUAGAACCACGG
miR-140-3p inhibitor	CCGUGGUUCUACCCUGUGGUA
NC	UACACCACGGGUAGGACGACA
siTYMS	UGUAUUCUGCCCCAAAAUGCC
siNC	UGUUAUGCCCCAAAACUCUGC

H23 and A549 cells at a density of 1 × 10^6^ cells/well were grown in 6-well plates until reached 80% confluence, and transfection was subsequently performed with Lipofectamine 3000 reagent (L3000-015, Invitrogen, Carlsbad, CA, USA) at room temperature according to the manuals of the producer. Cells were harvested 48 h after transfection.

### Dual-luciferase reporter (DLR) assay

Sequence of TYMS containing the target sites of miR-140-3p was synthesized and bought from GenePharma (Shanghai, China) and inserted into the pMirGLO luciferase vector (E1330, Promega, Madison, WI, USA) for wild-type reporter plasmids of TYMS (TYMS 3ʹ-UTR, 5ʹ-aucaugauguagagUGUGGUu-3ʹ). Mutagenesis was structured by a site-directed mutagenesis kit (11003ES10, Yeasen, Shanghai, China) for TYMS-3ʹ-UTR-mutated reporter plasmid (TYMS-3ʹ-UTR-MUT, 5ʹ-aucaugauguagagUAUCGUu-3ʹ).

H23 and A549 cells at a density of 5 × 10^3^ cells/well were grown in 48-well plates and subjected to the transfection with miR-140-3p or Control and reporter plasmid of TYMS-3ʹ-UTR and TYMS-3ʹ-UTR-MUT by Lipofectamine 3000 reagent at room temperature. 48 h later, H23 and A549 cells were transferred to luciferase activity detection under the DLR system (E1910, Promega, Madison, WI, USA) and SPECTROstar Nano microplate reader (BMG Labtech, Hopkinton, MA, USA) as per the protocols of the manufacturer. *Renilla* luciferase activity was utilized as the normalization of the firefly luciferase activity.

### Cell viability, migration, invasion, proliferation and angiogenesis assays

Cell Counting Kit-8 (CCK-8) assay was used for detecting the viability of transfected cells. Transfected H23 and A549 cells at a density of 2 × 10^3^ cells/well were inoculated in 96-well plates for 24 and 48 h at 37°C with 5% CO_2_. Ten microliters of CCK-8 reagent (G-020-1-1, Nanjing Jiancheng Bioengineering Institute (NJBI), Nanjing, China) was added to the cells and incubated for 4 h. The optical density (OD) value was recorded in an iMark Microplate Absorbance Reader (Bio-Rad, Hercules, CA, USA) at an absorbance of 450 nm.

For the Scratch assay to evaluate the migration capability of transfected cells, transfected H23 and A549 cells at a density of 1 × 10^5^ cells/well were maintained in 6-well plates. The man-made wound was created after cells were completely confluent and additionally cultured in serum-free medium at 37°C with 5% CO_2_. Cell images at 0 and 24 h were taken under an inverted microscope (Eclipse Ti2, Nikon, Tokyo, Japan) under the magnification of 100 × .

In addition, Transwell assay was adopted to determine the invasion of transfected cells. The pre-thawed 50 μL Matrigel (M8370, Solarbio, China) was coated onto the 8-μm-pore Transwell chambers, which were placed on 24-well plates. Then, 1 × 10^5^ transfected H23 and A549 cells with 200 μL non-serum medium were transferred to the upper Transwell chamber at 37°C with 5% CO_2_, and the corresponding lower chambers were added with 700 μL complete medium. Twenty-four hours later, the lower Transwell chambers were washed, fixed in paraformaldehyde (4%, P1110, Solarbio, China) for 30 min and stained by Giemsa (0.1%, G1015, Solarbio, China) for 20 min. Cells were finally numbered under an inverted microscope and photographed under 100 × magnification in five randomly picked areas.

For colony formation assay to determine the proliferation capability, transfected H23 and A549 cells at a density of 1 × 10^3^ cells/well were seeded in 6-well plates for 14 days (d). Cells were first fixed by methanol (M116122, Aladdin, Shanghai, China) for 15 min, and then stained using crystal violet (C8470, Solarbio, China) for 30 min. Visible colonies formed were observed under a digital camera (D500, Nikon, Tokyo, Japan). Images of the colony formation were photographed, followed by the calculation on the colony formation rates.

The angiogenesis assay were conducted as illustrated previously [20]. All cells, including LUAD cells and HUVECs, were washed using phosphate buffer saline (PBS, I010-1-1, NJBI, China) three times and detached using 0.05% trypsin/ethylenediaminetetraacetic acid (EDTA) (T3924, Sigma-Aldrich, USA). After centrifugation, all cell pellets were washed with PBS again and counted using a cell counter (#6749, Corning, Inc., Corning, NY, USA). Then, 24-well plates were coated with Matrigel and subsequently incubated with at 37°C for 30 min. And, 2 × 10^4^ cells were resuspended with EGM-2, and loaded onto the top of Matrigel, and treated with the conditioned medium from the cultured LUAD cells to evaluate the angiogenesis. After 4 h, the image of tube structures was captured using an inverted optical microscope under 200 × magnification. Data on tube length were quantified using Tube formation ACAS Image Analysis Software (v.1.0, ibidi GmbH, Gräfelfing, Germany).

### RNA isolation

Trizol (15596–018, Invitrogen, USA) was used for total RNA extraction from transfected or untransfected cells based on the protocol of the producer, and miRNA extraction kit (K1456, BioVision, Milpitas, CA, USA) was utilized to extract miRNA in line with the instructions provided by the manufacturers. The extracted RNA was preserved in −80°C, the concentration of which was determined by the NanoDrop Lite spectrophotometer (ND-LITE, Thermo Fisher Scientific, Waltham, MA, USA).

### Quantitative real-time polymerase chain reaction (qRT-PCR)

qRT-PCR was operated using a One-Step qRT-PCR kit (E5315S, New England BioLabs, Ipswich, MA, USA), and PCR was performed in a One-Step Quantitative RT-PCR System (11732–927, Applied Biosystems, Foster City, CA, USA) under these conditions as guided by the manufacturers: 1 cycle of reverse transcription at 48°C for 15 min, 1 cycle of initial denaturation at 94°C for 1 min, and total 40 cycles of denaturation at 94°C for 15 seconds (sec), annealing at 65°C for 30 sec and extension at 68°C for 1 min, followed by the final extension at 68°C for 5 min, with GAPDH and U6 as internal controls. Primer sequences are listed in Table 2. Relative expressions were finally quantified via the 2^−ΔΔCT^ method [21]. The component of PCR system was listed as follows: 25 μl of 2 × OneTaq One-Step Reaction Mix, 2 μl 25 × OneTaq One-Step Enzyme Mix, 2 μl of both forward and reverse primer, 2 μl of 1 μg total RNA, and 17 μl of RNase-free water.

**Table 2.** Primers for qRT-PCR

Gene	Primers (5ʹ–3ʹ)	
	Forward	Reverse
miR-192-5p	TTGACAGCCGTCGTATCCAG	GTATCCAGTGCGTGTCGTGG
miR-140-3p	CGGGTCGTATCCAGTGCAAT	GTCGTATCCAGTGCGTGTCG
miR-215-5p	AGACGTCGTATCCAGTGCAA	GTCGTATCCAGTGCGTGTCG
TYMS	CAGATCCAACACATCCTC	CCTTCAATCTGAAAGTCTTC
E-cadherin	CCCGGGACAACGTTTATTAC	GCTGGCTCAAGTCAAAGTCC
N-cadherin	GCTCTCCCTCCCTGTTCC	GGACTCGCACCAGGAGTAATAA
Vimentin	CTTAAAGGAACCAATGAGTC	GAGAAGTTTCGTTGATAACC
U6	CTCGCTTCGGCAGCACATATACT	ACGCTTCACGAATTTGCGTGTC
GAPDH	TTTTTGGTTTTAGGGTTAGTTAGTA	AAAACCTCCTATAATATCCCTCCTC

### Western blot

Protein expressions were determined based on a previous study [22]. After the collection of cells, the lysis and extraction of the protein were performed using RIPA lysis buffer (R0010), and the concentration was determined with Bicinchoninic acid (BCA) protein kit (PC0020). 20 μg samples of protein lysates were firstly electrophoresed and detached via sodium dodecyl sulfate-polyacrylamide gel electrophoresis (SDS-PAGE, P1200), and transferred onto polyvinylidene fluoride (PVDF) membrane (YA1701) which was blocked using skimmed milk (5%) for 2 h and incubated with those primary antibodies against TYMS, E-cadherin, N-cadherin, vimentin, vascular endothelial growth factor (VEGF), cleaved caspase-3, caspase-3, B-cell lymphoma 2 (Bcl-2), Bcl-2-associated X protein (Bax) at 4°C overnight with internal control GAPDH. After that, the membrane was incubated with secondary antibodies at room temperature for 1 h and washed using tris-buffer saline tween (TBST, T196393, Aladdin, China) for three times. After collecting protein band, enhanced chemiluminescence (ECL) kit (SW2020) was employed for visualization. Data on the strips were analyzed in iBright CL1500 Imaging System (A44240, Invitrogen, USA) and the gray values were calculated using ImageJ (v. 5.0, Bio-Rad, USA). All reagents used here were purchased from Solarbio except for those indicated and the operations with the reagent were guided as the protocols of the manufacturer. All information of antibodies was available in Table 3.

**Table 3.** Antibodies for Western blot

Antibody	Host	Catalog no.	Dilution	Brand
Anti-TYMS antibody	Rabbit	ab108995	1:1000	Abcam
Anti-E-cadherin antibody	Rabbit	ab40772	1:10,000	Abcam
Anti-N-cadherin antibody	Rabbit	ab18203	1:1000	Abcam
Anti-vimentin antibody	Rabbit	ab92547	1:2000	Abcam
Anti-VEGF antibody	Mouse	ab1316	1:1000	Abcam
Anti-Bcl-2 antibody	Rabbit	ab59348	1:1000	Abcam
Anti-Bax antibody	Rabbit	ab32503	1:10,000	Abcam
Anti-cleaved caspase-3 antibody	Rabbit	ab2302	1:2000	Abcam
Anti-caspase-3 antibody	Rabbit	ab32351	1:2000	Abcam
Anti-GAPDH antibody	Mouse	ab8245	1:2000	Abcam
Goat anti-rabbit IgG H&L	Goat	ab205718	1:2000	Abcam
Goat anti-mouse IgG H&L	Goat	ab205719	1:2000	Abcam

### Statistical analyses

All the statistics were analyzed using SPSS (v. 19.0, SPSS, Chicago, IL, USA), and each experiment was conducted in over three times independently, from which all data were expressed as mean ± standard deviation (SD). Statistical significances, determined by one-way ANOVA followed by Bonferroni post hoc test, was defined when *P* < 0.05.

## Results

In this study, we mainly explored the roles and functions of miR-140-3p and TYMS in LUAD, with the hope to work out a possible treatment strategy for LUAD in future clinical practice. We found that miR-140-3p inhibited the proliferation, migration, invasion and angiogenesis of lung adenocarcinoma cells via targeting TYMS.

### TYMS was high-expressed yet miR-140-3p was low-expressed in LUAD cells

**Figure 1.**
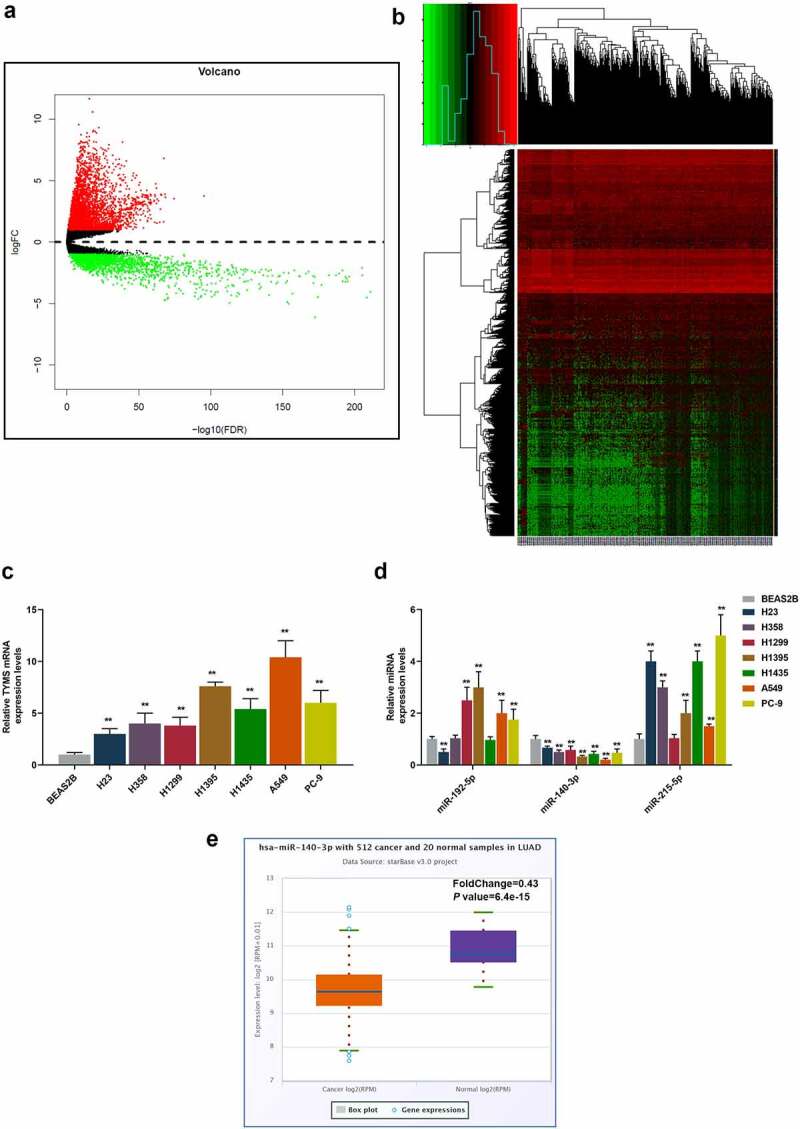
TYMS expression was upregulated yet miR-140-3p expression was downregulated in LUAD cells. (a–b) Bioinformatic analysis using the data retrieved from The Cancer Genome Atlas (https://www.genome.gov/Funded-Programs-Projects/Cancer-Genome-Atlas) was utilized to predict the candidate gene for our study. (c–d) Relative mRNA expression of TYMS (c) in bronchial epithelial cells BEAS2B and LUAD cells (H23, H358, H1299, H1395, H1435, A549 and PC-9) was quantified using qRT-PCR. GAPDH was the internal control. (d) Relative expression of candidate miRNA which could target TYMS, including miR-192-5p, miR-140-3p and miR-215-5p, in bronchial epithelial cells BEAS2B and LUAD cells (H23, H358, H1299, H1395, H1435, A549 and PC-9) was detected by qRT-PCR. U6 was employed as internal controls. (e) Bioinformatic analysis from starBase (http://starbase.sysu.edu.cn) confirmed the expression of miR-140-3p in LUAD. All experiments have been performed in triplicate and data were expressed as mean ± standard deviation (SD). **P < 0.001, vs. BEAS2B. TYMS: thymidylate synthetase; miR: miRNA; LUAD: lung adenocarcinoma; qRT-PCR: quantitative real-time polymerase chain reaction

In the beginning, with the help of bioinformatic analysis, we successfully predicted TYMS as the candidate gene for our study (Figure 1a-b). Then, to confirm the role of TYMS played in LUAD, TYMS expression in LUAD and bronchial epithelial cells BEAS2B was respectively measured. Compared to BEAS2B cells, TYMS expression was upregulated but varied in LUAD cells, with the highest expression present in A549 cells and the lowest expression present in H23 cells (Figure 1c, *P* < 0.001). Then, we used bioinformatic analyses to predict the potential miRNAs that could target TYMS, among which miR-140-3p aroused our interest, as only miR-140-3p expression was downregulated in all LUAD cells in comparison with bronchial epithelial cells BEAS2B. It was observed that among these LUAD cells, the highest expression of miR-140-3p was found in the H23 cell and lowest expression in A549 cell (Figure 1d, *P* < 0.001). H23 and A549 cells were thus chosen for subsequent studies. Furthermore, the bioinformatic analysis from starBase confirmed the low-expressed miR-140-3p in LUAD (Figure 1e).

### Upregulation of miR-140-3p promoted miR-140-3p yet suppressed TYMS in LUAD cells, while downregulation of miR-140-3p did conversely

**Figure 2.**
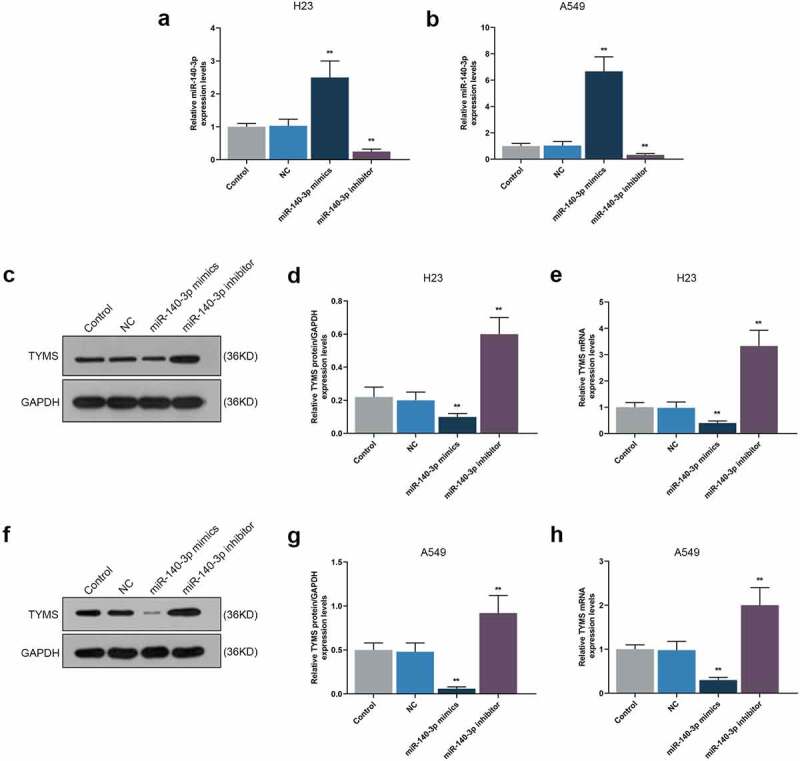
Upregulation of miR-140-3p promoted miR-140-3p expression yet suppressed TYMS expression in LUAD cells, while downregulation of miR-140-3p did the opposite. (a–b) Relative mRNA expression of miR-140-3p in LUAD cells H23 (a) and A549 (b) following miR-140-3p mimic or inhibitor transfection was determined by qRT-PCR. U6 was chosen as internal reference. (c–h) Relative protein/GAPDH and mRNA expression of TYMS in LUAD cells H23 (c–e) and A549 (f–h) following miR-140-3p mimic or inhibitor transfection were measured by Western blot and qRT-PCR. GAPDH was used as internal control. All experiments have been performed in triplicate and data were expressed as mean ± standard deviation (SD). **P < 0.001, vs. NC. NC: negative control

To detect the effect of miR-140-3p on TYMS expression in LUAD cells, both miR-140-3p mimic and inhibitor were transfected into H23 and A549 cells. miR-140-3p and TYMS expressions were measured, the results of which suggested that miR-140-3p mimic was associated with the evident increase of miR-140-3p expression and the decrease of TYMS expression (Figure 2a-h, *P* < 0.001). On the contrary, miR-140-3p expression was decreased yet TYMS expression was increased induced by miR-140-3p inhibitor (Figure 2a-h, *P* < 0.001).

### Upregulation of miR-140-3p repressed the viability, proliferation, migration, invasion and angiogenesis of LUAD cells, while downregulation of miR-140-3p did the opposite

**Figure 3.**
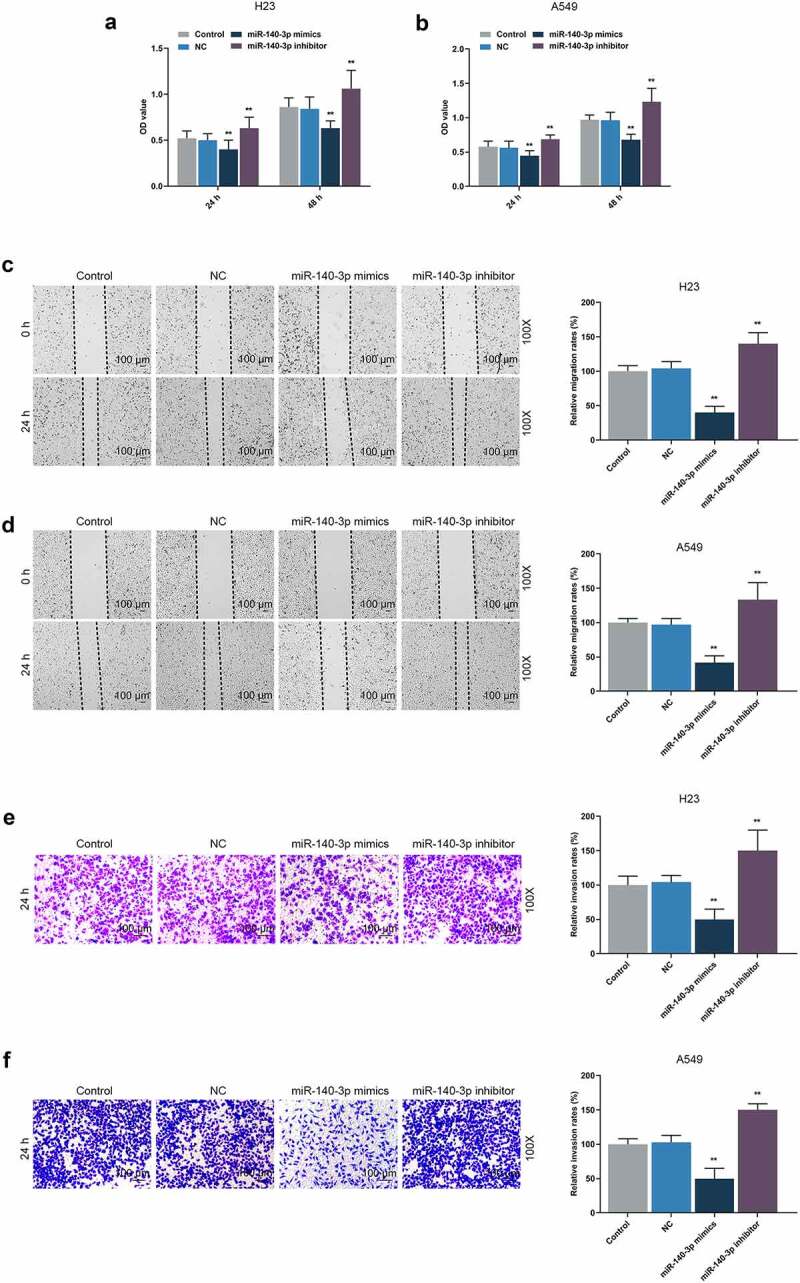
Upregulation of miR-140-3p suppressed the viability, migration and invasion of LUAD cells, while downregulation of miR-140-3p did the opposite. (a–b) The viability of LUAD cells H23 (a) and A549 (b) after miR-140-3p mimic or inhibitor transfection at 24 and 48 h was determined by CCK-8 assay. (c-d) Relative migration rate of LUAD cells H23 (c) and A549 (d) after miR-140-3p mimic or inhibitor transfection was measured by Scratch assay at 0 and 24 h, under 100 × magnification. (e–f) Relative invasion rate of LUAD cells H23 (e) and A549 (f) after miR-140-3p mimic or inhibitor transfection was measured by Transwell assay at 24 h, under 100 × magnification. All experiments have been performed in independent triplicate and data were expressed as mean ± standard deviation (SD). **P < 0.001, vs. NC. CCK-8: Cell Counting Kit-8

CCK-8 assay was used for detecting the effects of miR-140-3p on the viability of LUAD cells. At both 24 and 48 h, a decreased OD value was found after upregulation of miR-140-3p, whereas downregulation of miR-140-3p led to an increased OD value (Figure 3a-b, *P* < 0.001), presenting that upregulation of miR-140-3p could suppress LUAD cells viability, whereas downregulation of miR-140-3p elicited a contrary result.

**Figure 4.**
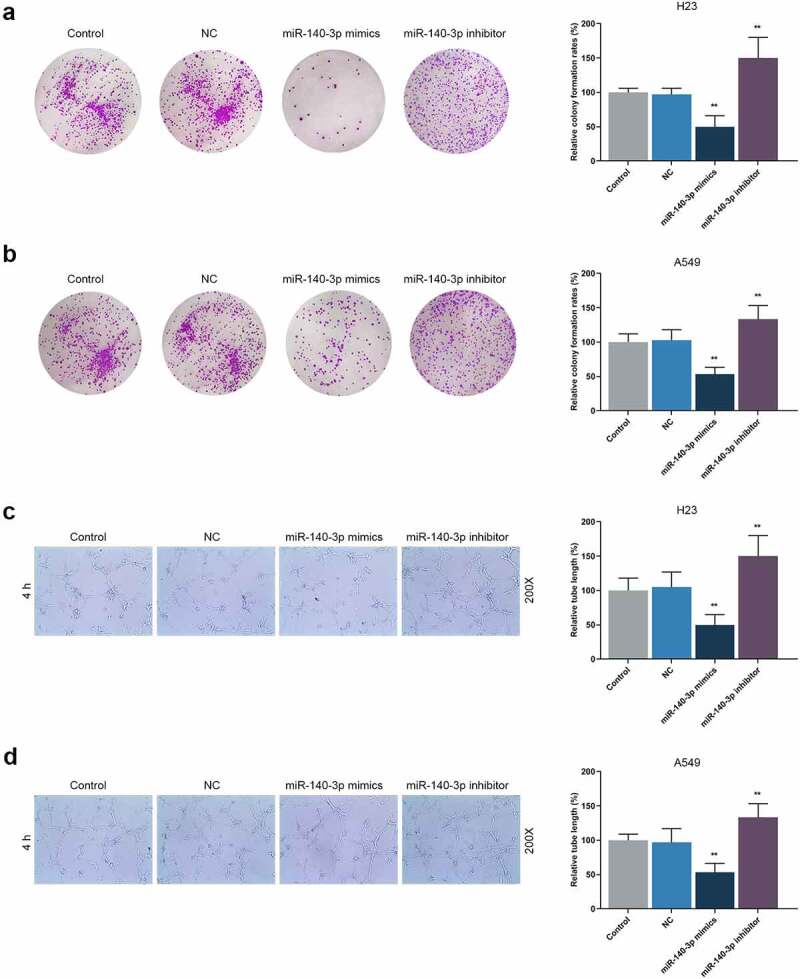
Upregulation of miR-140-3p suppressed the proliferation and tube formation of LUAD cells, while downregulation of miR-140-3p did the opposite. (a–b) Relative colony formation rate of LUAD cells H23 (a) and A549 (b) after miR-140-3p mimic or inhibitor transfection was detected by colony formation assay. (c–d) Relative tube lengths of LUAD cells H23 (c) and A549 (d) after miR-140-3p mimic or inhibitor transfection were measured with tube formation at 4 h, under 200 × magnification. All experiments have been performed in independent triplicate and data were expressed as mean ± standard deviation (SD). **P < 0.001, vs. NC

According to Scratch assay and Transwell assay, upregulation of miR-140-3p reduced the migration and invasion of LUAD cells, while downregulation of miR-140-3p promoted the migration and invasion of LUAD cells (Figure 3c-f, *P* < 0.001). As depicted in the results from colony formation assay and tube formation assay, upregulation of miR-140-3p caused decreases of colony formation and tube length, while downregulation of miR-140-3p was associated with an increased colony formation and tube length (Figure 4a-d, *P* < 0.001).

### TYMS was the target gene of miR-140-3p

**Figure 5.**
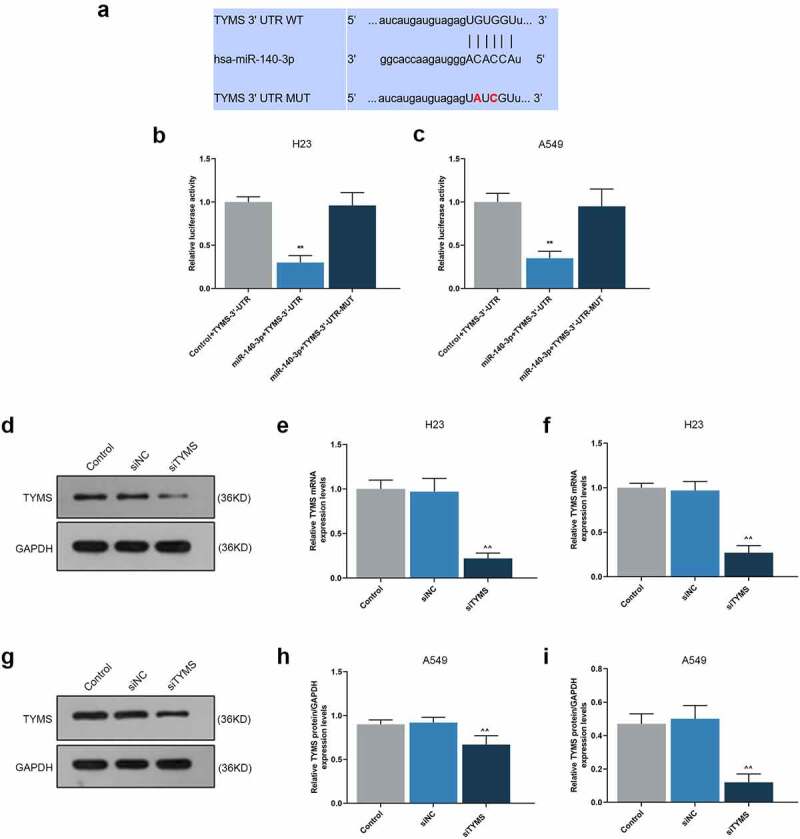
TYMS was the target gene of miR-140-3p, and silencing TYMS caused a decrease on TYMS expression in LUAD cells. (a) Conserved binding sites between TYMS and miR-140-3p were predicted by starBase. (b–c) Results from DLR assay suggested that TYMS was the target gene of miR-140-3p. (d–i) Relative protein/GAPDH and mRNA expressions of TYMS in LUAD cells H23 (d–f) and A549 (g–i) after transfection of siTYMS were determined by Western blot and qRT-PCR. GAPDH was used as an internal control. All experiments were performed in triplicate and data were expressed as mean ± standard deviation (SD). **P < 0.001, vs. Control+TYMS-3ʹ-UTR; ^^^^*P* < 0.001, vs. siNC. DLR: dual-luciferase reporter; 3ʹ-UTR: 3ʹ-untranslated region; WT: wild-type; MUT: mutation type; siTYMS: small-interfering RNA for TYMS

Using starBase, we confirmed TYMS as the target gene of miR-140-3p, and the conserved binding sites between these two were shown in Figure 5a. Then, the confirmation via DLR assay suggested that when compared with Control+TYMS-3ʹ-UTR group, the luciferase activity in miR-140-3p+TYMS-3ʹ-UTR group was decreased (Figure 5b-c, *P* < 0.001), while no significant changes of that were evidenced in miR-140-3p+TYMS’-3ʹ-UTR-MUT group. These results indicated that TYMS was the target gene of miR-140-3p.

### Silenced TYMS reversed the effects of downregulation of miR-140-3p in LUAD cells

To unearth the role and effect of TYMS on LUAD cells, siTYMS was transfected into LUAD cells, and it was displayed that TYMS expression was downregulated after siTYMS transfection (Figure 5d-i, *P* < 0.001), indicating that silenced TYMS resulted in decreased TYMS expression in LUAD cells.

**Figure 6.**
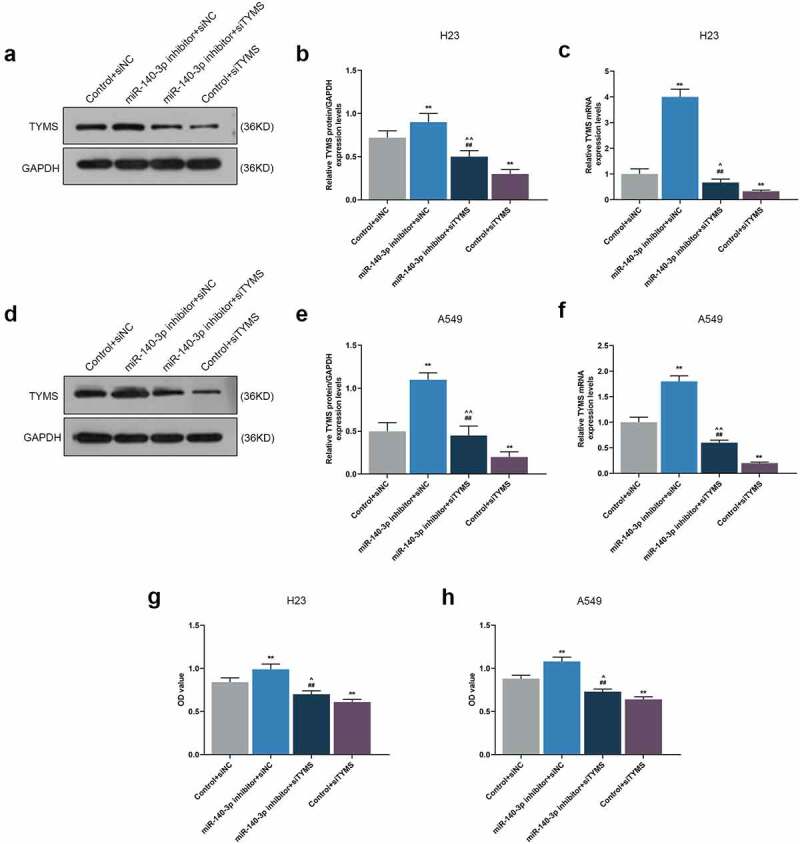
Silenced TYMS reversed the effects of downregulation of miR-140-3p on TYMS expression and cell viability in LUAD cells. (a–f) Relative protein/GAPDH and mRNA expression of TYMS in LUAD cells H23 (a–c) and A549 (d–f) after miR-140-3p inhibitor and siTYMS transfection were detected by Western blot and qRT-PCR. GAPDH was used as internal control. (g–h) The viability of LUAD cells H23 (g) and A549 (h) after miR-140-3p inhibitor and siTYMS transfection was determined by CCK-8 assay. All experiments have been performed in independent triplicate and data were expressed as mean ± standard deviation (SD). **P < 0.001, vs. Control+siNC; ^##^*P* < 0.001, vs. miR-140-3p inhibitor+siNC; ^^^*P* < 0.05, ^^^^*P* < 0.001, vs. Control+siTYMS

To further determine the effects and interactions between miR-140-3p and TYMS on LUAD cells, miR-140-3p inhibitor and siTYMS were transfected into LUAD cells, and we measured TYMS expression in LUAD cells. After downregulation of miR-140-3p, TYMS expression was upregulated, whereas silenced TYMS resulted in a decreased TYMS expression (Figure 6a-f, *P* < 0.001). Silenced TYMS was further confirmed to reverse the effects of downregulation of miR-140-3p on TYMS expression in LUAD cells (Figure 6a-f, *P* < 0.05).

**Figure 7.**
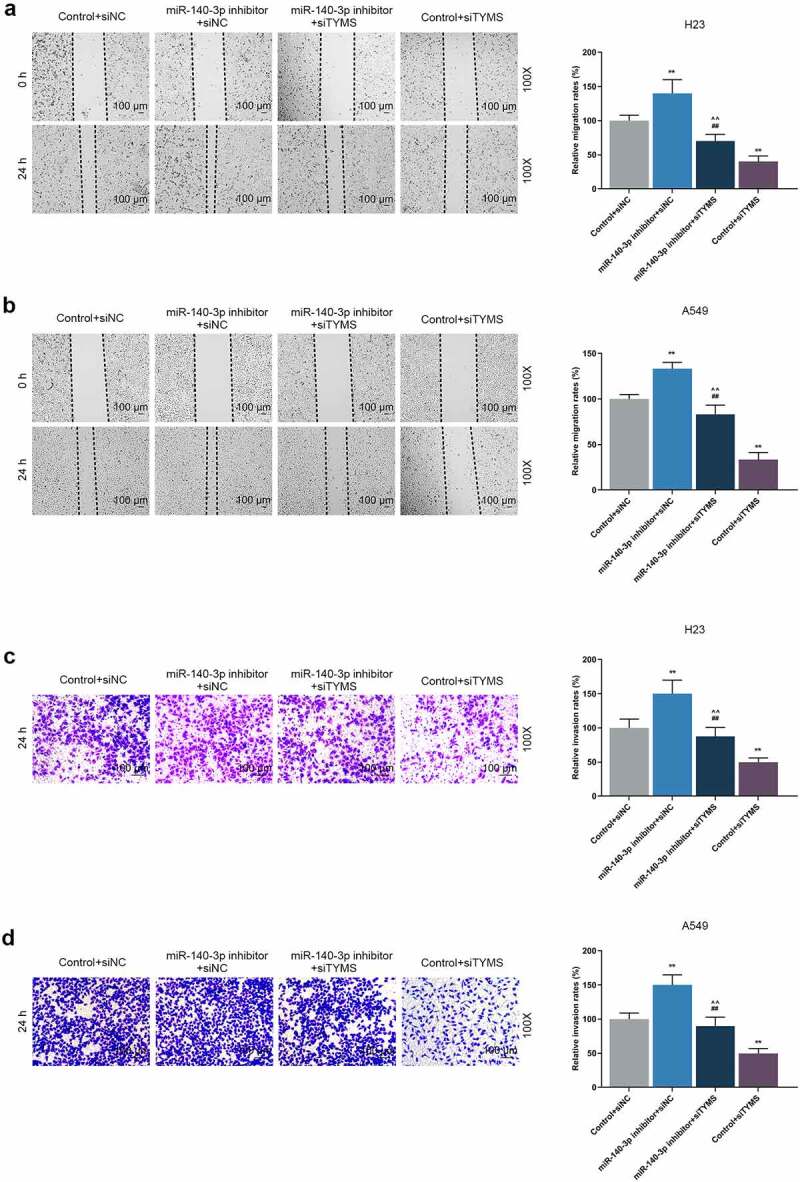
Silenced TYMS reversed the effects of downregulation of miR-140-3p on LUAD cells migration and invasion. (a–b) Relative migration rate of LUAD cells H23 (a) and A549 (b) after miR-140-3p inhibitor and siTYMS transfection was measured by Scratch assay at 0 and 24 h, under 100 × magnification. (c–d) Relative invasion rate of LUAD cells H23 (c) and A549 (d) after miR-140-3p inhibitor and siTYMS transfection was measured by Transwell assay at 24 h, under 100 × magnification. All experiments have been performed in independent triplicate and data were expressed as mean ± standard deviation (SD). *P < 0.05, **P < 0.001, vs. Control+siNC; ^##^*P* < 0.001, vs. miR-140-3p inhibitor+siNC; ^^^*P* < 0.05, ^^^^*P* < 0.001, vs. Control+siTYMS

As can be noted from CCK-8 assay, the OD value of LUAD cells was increased by downregulation of miR-140-3p but reduced by silencing of TYMS (Figure 6g-h, *P* < 0.001). Meanwhile, silencing of TYMS reversed the effects of downregulation of miR-140-3p on the viability of LUAD cells (Figure 6g-h, *P* < 0.05). After downregulation of miR-140-3p, the migration and invasion of LUAD cells were both upregulated, whereas silencing of TYMS generated inverse effects (Figure 7a-b, *P* < 0.001; Figure 7c-d, *P* < 0.001). Meanwhile, silenced TYMS reversed the effects of downregulation of miR-140-3p on the migration and invasion of LUAD cells (Figure 7a-d, *P* < 0.001).

**Figure 8.**
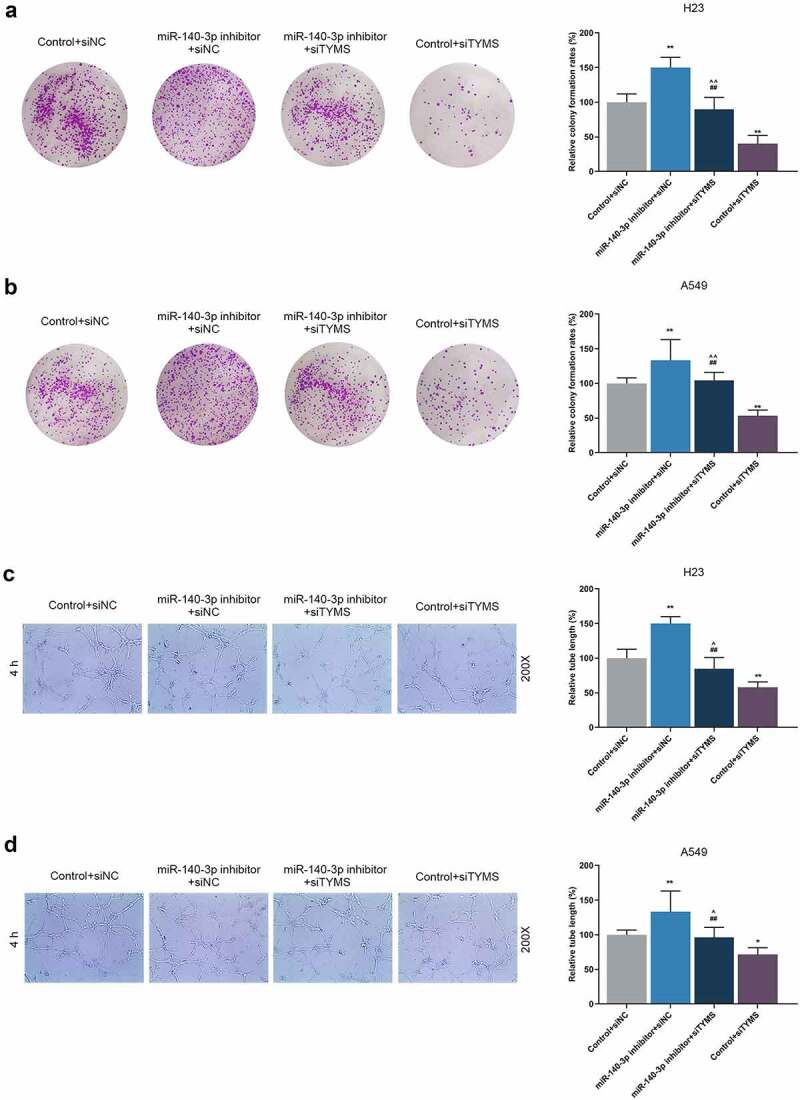
Silenced TYMS reversed the effects of downregulation of miR-140-3p on LUAD cells proliferation and tube formation. (a–b) Relative colony formation rate of LUAD cells H23 (a) and A549 (b) after miR-140-3p inhibitor and siTYMS transfection was detected by colony formation assay. (c–d) Relative tube lengths of LUAD cells H23 (c) and A549 (d) after miR-140-3p inhibitor and siTYMS transfection were measured with tube formation at 4 h, under 200 × magnification. All experiments have been performed in independent triplicate and data were expressed as mean ± standard deviation (SD). *P < 0.05, **P < 0.001, vs. Control+siNC; ^##^*P* < 0.001, vs. miR-140-3p inhibitor+siNC; ^^^*P* < 0.05, ^^^^*P* < 0.001, vs. Control+siTYMS

It was concluded from the illustrated results of colony formation and tube formation assay that after downregulation of miR-140-3p, the colony formation in LUAD cells and tube length were promoted, whereas those were reduced by silenced TYMS (Figure 8a-b, *P* < 0.001; Figure 8c-d, *P* < 0.05). Also, silenced TYMS abrogated the effects of downregulation of miR-140-3p on the colony formation in LUAD cells and tube length (Figure 8a-b, *P* < 0.001; Figure 8c-d, *P* < 0.05).

### Silenced TYMS reversed the effects of downregulation of miR-140-3p on epithelial-to-mesenchymal transition, apoptosis and angiogenesis-related proteins in LUAD cells

**Figure 9.**
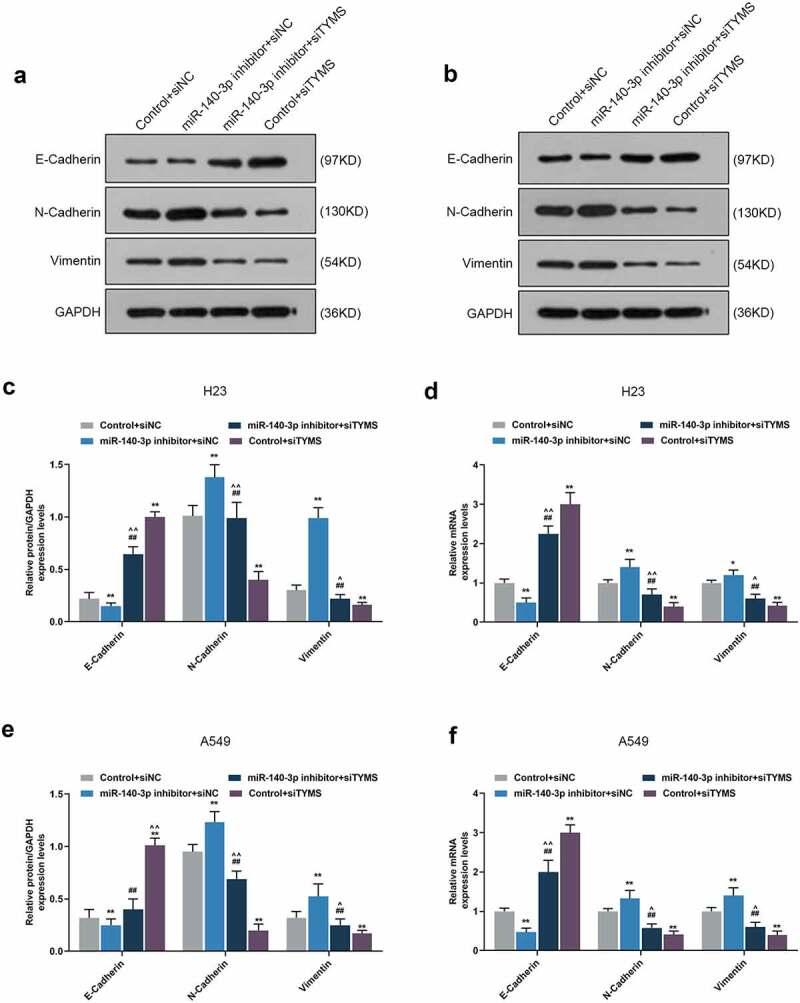
Silenced TYMS reversed the effects of downregulation of miR-140-3p on epithelial-to-mesenchymal transition-related proteins expressions in LUAD cells. (a–f) Relative protein/GAPDH and mRNA expressions of E-cadherin, N-cadherin and vimentin in LUAD cells H23 and A549 after miR-140-3p inhibitor and siTYMS transfection were measured by Western blot and qRT-PCR. GAPDH was chosen as internal control. All experiments have been performed in triplicate and experimental data were expressed as mean ± standard deviation (SD). *P < 0.05, **P < 0.001, vs. Control+siNC; ^##^*P* < 0.001, vs. miR-140-3p inhibitor+siNC; ^^^*P* < 0.05, ^^^^*P* < 0.001, vs. Control+siTYMS

**Figure 10.**
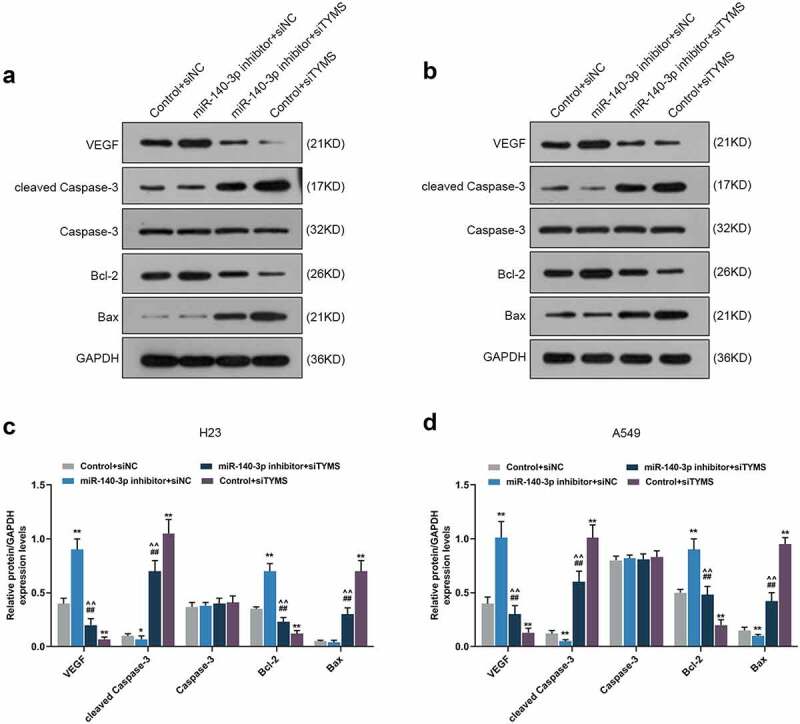
Silenced TYMS reversed the effects of downregulation of miR-140-3p on apoptosis and angiogenesis-related proteins expressions in LUAD cells. (a–d) Relative protein/GAPDH expression of VEGF, cleaved caspase-3, caspase-3, Bcl-2 and Bax in LUAD cells H23 and A549 after miR-140-3p inhibitor and siTYMS transfection was measured by Western blot. GAPDH was chosen as internal control. All experiments have been performed in triplicate and experimental data were expressed as mean ± standard deviation (SD). *P < 0.05, **P < 0.001, vs. Control+siNC; ^##^*P* < 0.001, vs. miR-140-3p inhibitor+siNC; ^^^^*P* < 0.001, vs. Control+siTYMS. VEGF: vascular endothelial growth factor; Bcl-2: B-cell lymphoma 2; Bax: Bcl-2-associated X protein

In LUAD cells, after downregulation of miR-140-3p, N-cadherin, vimentin, VEGF and Bcl-2 expressions were upregulated, yet E-cadherin, cleaved caspase-3 and Bax expressions were downregulated, whereas silenced TYMS had the opposite effects (Figure 9a-f, *P* < 0.05; Figure 10a-d, *P* < 0.05). Also, silencing TYMS reversed the effects of downregulation of miR-140-3p in LUAD cells (Figure 9a-f, *P* < 0.05; Figure 10A-d, *P* < 0.05).

## Discussion

Increasing evidence has unveiled the regulation of miRNAs on cell proliferation, metastasis and apoptosis, and when it comes to miR-140-3p, mounting discoveries have provided the evidence concerning the participation of miR-140-3p in the suppression of non-small cell lung cancer (NSCLC), which can be further classified into LUAD and lung squamous cell carcinoma (LUSC) [14,23–25]. We, here, confirmed that the upregulation of miR-140-3p had inhibitory effects on the viability, proliferation, migration, invasion and angiogenesis of LUAD cell via targeting TYMS, which not only provides empirical evidence regarding the participation and suppression of miR-140-3p in LUAD but also proves for the first time the interactions of both miR-140-3p and TYMS in LUAD.

As mentioned above, the suppressive effects of miR-140-3p in LUAD have been addressed, with the discussion on the progression and stem cell-like properties in LUAD [13,14]. These discoveries have thus laid solid foundation for our study, which additionally evidences the suppressive role of miR-140-3p in LUAD. The proliferation has been uncovered as an essential part for cancer development and progression, while metastasis, where malignant cells migration and invasion play key roles, symbolizes an advanced stage of malignancy, which comes up as the leading factor of cancer-related death [26,27]. Upregulation of miR-140-3p is suggested to be not only associated with the suppression on the biological behaviors of LUAD cells, including proliferation, migration, and invasion but also related to the reversion on the promotive effects of circRNA AASDH (circAASDH) on LUAD cells [14]. We, likewise, discovered that miR-140-3p upregulation resulted in suppressive effects on the viability, proliferation, migration and invasion of LUAD cells, and took initiative to validate the downregulation of miR-140-3p caused the decrease of E-cadherin yet the increase of both N-cadherin and vimentin in LUAD cells, which was similar to the results of a prior discovery that the upregulated miR-140-3p was associated with the reduced N-cadherin and vimentin expressions yet promoted E-cadherin expression in hepatocellular carcinoma (HCC) cells [28]. E-cadherin, a glycoprotein with transmembrane property, has been identified as pivotal in cell–cell adhesion and has been recognized to act as an inhibitor in malignancies, while N-cadherin, on the contrary, endows malignant cells with promotive effect on metastasis, and the acquisition of N-cadherin in tumor cells is regarded as a vital step in epithelial cancer metastasis [29,30]. Vimentin is a cytoskeletal protein to compose intermediate filament and is of great significance in epithelial-to-mesenchymal transition (EMT), and vimentin overexpression is identified and recognized as a prerequisite for the metastasis of human cancers [31]. Defined as new vessels formation, angiogenesis is central in growth of cancer cells and metastasis of tumor, where VEGF, a glycoprotein with homo-dimeric property acting as an essential mediator for angiogenesis in cancer as demonstrated previously, is indispensable for cancer growth and development [32,33]. It was disclosed that the upregulation of miR-140-3p decreases the expression of VEGF in CRC cells, which is associated with the aggressiveness of CRC, as well as the discovery, which proves that the silenced miR-140-3p restrains the angiogenesis in endothelial cells (ECs), while the overexpressed miR-140-3p did conversely [34]. To the best of our knowledge, we put forward for the first time that the upregulation of miR-140-3p decreased the angiogenesis, along with the suppression on VEGF expression in LUAD cells, indicating that miR-140-3p upregulation could suppress the angiogenesis. Altered apoptosis has been seen to take responsibility not only for tumor development and progression but also for tumor resistance against therapies and treatment. Prior studies have suggested that the increased caspase-3 activity in LUAD cells A549 and Calu3 is associated with the upregulation of miR-140-3p, while the decreased Bcl-2 is associated with the elevation of Bax and cleaved caspase-3 in cervical cancer cell Caski [13,35]. In the light of our study, miR-140-3p downregulation was found to be related to the decrease of cleaved caspase-3 and Bax and the increase of Bcl-2 in LUAD cells, all of which were unveiled as apoptosis-associated factors [36–38]. However, the underlying molecular mechanisms needed to be further addressed in detail.

The replication of all cells needs thymidine, a pyrimidine which makes up DNA and synthesized in human cells *de novo*, and the last step of the synthesis is catalyzed by the enzyme TYMS, which is coded by the thyA gene and is the best-known target in anti-cancer therapy as a protein with catalytic and regulatory functions [39,40]. As a class of methyltransferase enzymes, TYMS is pivotal for the survival and replication of cells as it can provide the unique biosynthetic source of 2ʹ-deoxythymidine-5ʹ-monophosphate (dTMP), which is of great essence for DNA synthesis [41]. TYMS has been proved to be the downstream target gene of miR-375-3p, which enhances the chemosensitivity of CRC cells to 5-fluorouracil (5-Fu) [42]. Also, when it comes to LUAD, TYMS has been sorted as one of the differentially expressed metabolism-related genes (MRGs), which provides the evidence on the possible role of TYMS in LUAD [43]. However, its interaction with miR-140-3p, to be precisely, remained poorly understood. We further expanded the role of TYMS in LUAD. Specifically, we first confirmed TYMS, which was upregulated in LUAD, as the target gene of miR-140-3p. Besides, silenceing TYMS not only inhibited the viability, proliferation, migration, invasion and angiogenesis but also reversed the promotive effect of miR-140-3p downregulation in LUAD cells, with the upregulation on E-cadherin, cleaved caspase-3 and Bax and the downregulation on N-cadherin, vimentin, VEGF, and Bcl-2, the results of which further completed the role of TYMS in LUAD. With the development of biotechnology, increasing attention has been paid to the research of cancer, for example, Sudhakara et al. reported a low-cost electrochemical immunosensor for quantitative early detection of pancreatic cancer biomarker PEAK1 [44]. More diagnostic methods and biomarkers of LUAD need to be further studied.

Nevertheless, there are some shortages in the current study that waited to be addressed. We solely figured out the effects of miR-140-3p and TYMS in LUAD cells *in vitro*. However, a corresponding validation *in vivo* was required to validate the result. In addition, despite the confirmed expression changes on apoptosis-associated genes in LUAD cells, the specific effects of miR-140-3p and TYMS on the apoptosis in LUAD cells remain vague. Future research, consequently, is required to improve and perfect the results in our study.

## Conclusion

We unraveled that upregulation of miR-140-3p, a miRNA low-expressed in LUAD, led to decreased cell viability, proliferation, migration, invasion and angiogenesis, which could be achieved via targeting TYMS. Our present study also provided another evidence of miR-140-3p and the first evidence concerning the interaction between miR-140-3p and TYMS in LUAD. It is hoped that the results proposed in our current study can contribute to bringing further insights into the roles of miRNAs in the development and progression of LUAD and to providing possibly viable therapeutic methods for LUAD in future clinical practice.

## Data Availability

The analyzed data sets generated during the study are available from the corresponding author on reasonable request.

## Highlights

- MiR-140-3p was low-expressed, yet TYMS was high-expressed in LUAD cells.
- TYMS was the target gene of miR-140-3p.
- MiR-140-3p inhibited the malignant process of LUAD cells by targeting TYMS.
